# Stakeholders’ views on public-private partnerships for rehabilitation services in South Africa ahead of National Health Insurance

**DOI:** 10.4102/safp.v67i1.6013

**Published:** 2025-04-24

**Authors:** Senzelwe M. Mazibuko, Pragashnie Govender, Thayananthee Nadasan

**Affiliations:** 1 Discipline of Physiotherapy, School of Health Sciences, University of KwaZulu-Natal, Durban, South Africa; 2 eJimini Physiotherapists, Empangeni, South Africa; 3 Discipline of Occupational Therapy, School of Health Sciences, University of KwaZulu-Natal, Durban, South Africa

**Keywords:** availability, health access, National Health Insurance, public-private partnerships, quality of health, rehabilitation services, stakeholder perspectives

## Abstract

**Background:**

Quality rehabilitation services are limited in rural South African areas, such as KwaZulu-Natal (KZN). Public-private partnerships (PPPs) are increasingly valued as an effective model for public health delivery in developing countries; yet, their application in South Africa’s rehabilitation sector, especially with the upcoming National Health Insurance (NHI) for universal health coverage, remains unclear. This study examined perspectives on using PPPs for rehabilitation services within the District Health System in KZN in preparation for the NHI.

**Methods:**

A qualitative study in eThekwini, Amajuba and King Cetshwayo districts of KZN included 57 participants, selected through purposive sampling. Participants were rehabilitation practitioners, managers and social development representatives. Data were collected via focus groups and interviews and analysed using thematic analysis.

**Results:**

Participants noted that the NHI’s strategy of incorporating the private sector is designed to alleviate government pressure and provide financial incentives. Challenges mentioned include service availability, patient care and tariff disputes. Discrepancies between public and private sectors and the private sector’s financial sensitivities pose significant challenges to NHI implementation. A knowledge gap exists regarding the role of PPPs in the NHI context for rehabilitation services. Practitioners emphasised the necessity of adequate government funding for private sector partnerships to strengthen public health infrastructure.

**Conclusion:**

Stakeholders express varied views on PPPs for rehabilitation, highlighting the need for clear guidelines and funding support as South Africa nears NHI implementation.

**Contribution:**

This study provides insights into stakeholders’ views on PPPs for rehabilitation, identifying key benefits and challenges to inform effective NHI-aligned implementation strategies.

## Introduction

Access to rehabilitation services in lower- to middle-income countries such as South Africa has been challenging.^1,2,3,4,5^ Rehabilitation is an essential health service that improves the lives of people with a wide range of health conditions, including many with disabilities, by supporting their ability to carry out daily activities.^6^ South Africa, as a signatory of the United Nations Convention on the Rights of People with Disabilities (UNCRPD), has developed the Framework and Strategy for Disability and Rehabilitation Services in South Africa (FSDRSA).^7^ The 2030 Agenda for Sustainable Development acknowledged that rehabilitation is an integral component of the healthcare continuum of service.^8^ Furthermore, the United Nations’ Political Declaration of the High-Level Meeting of the General Assembly on the Prevention and Control of Non-communicable Diseases emphasised the importance of rehabilitation across the life course, given the often-chronic nature of the diseases.^8^

South Africa has pursued universal health coverage (UHC) for its population since the first democratic elections in 1994.^9^ South Africa has undertaken many steps to achieve universal access to quality health care services in the Republic in accordance with section 27 of the Constitution, such as establish a National Health Insurance (NHI) Fund and set out its powers, function and governance structures, provide a framework for the strategic purchasing of health care services by the Fund on behalf of the users, create mechanisms for equitable, effective and efficient utilisation of the resources of the fund to meet the health needs of the population, preclude or limit undesirable, unethical and unlawful practices in relation to the Fund and its users, and provide for matters connected herewith.^10^

The *National Health Insurance Act* (Act No. 20 of 2023) was signed into law by the President Cyril Ramaphosa in May 2024.^10^ Addressing historical disparities and taking reasonable measures, within available resources, to progressively realise South Africans‘ right to access good quality, essential healthcare is enshrined in the Constitution of the Republic of South Africa, 1996.^9,10^ National Health Insurance intends to create a single framework throughout the Republic for public funding and public purchasing of health care services, medicines, health goods and health-related products, as well as to eliminate the fragmentation of healthcare funding in the Republic.^10^

Although NHI has been viewed as a social and moral imperative, it lacks clarity and insight into the opportunity to engage in the NHI policy formation.^11^

Pitfalls have been cited in current organisational operations; national and provincial governments continue to function in the detached and rigid top-down hierarchy. There is a need for training and development in the NHI policy formulation so that implementation can be monitored.^11^ The NHI is in the early phase of development and rehabilitation professionals need to engage and respond proactively.^12^ Poor financial management and low levels of provincial and national economic growth over the recent coronavirus disease 2019 (COVID-19) pandemic indicate that rehabilitation may slip further down the list of priorities by policymakers unless there is committed, unified action to the trajectory.^6^ South Africa also has several national policies that ensure all its citizens’ right to equitable access to quality healthcare and support. These policies include the Batho Pele (People First) Principles, the Department of Public Services and the FSDR in South Africa.^13^ Key clauses within these policies highlight that health services should be non-discriminatory, varied and flexible, and all necessary steps should be taken to include affected populations and local communities in the design and implementation of services.^14^

According to the South African Department of Finance (National Treasury), as guided by the *Public Financial Management Act* (PFMA), a public-private partnership (PPP) is an agreement between a government institution and a private party, where the latter assumes significant financial, technical and operational risk.^15^ Private partners shoulder risk by designing, constructing and managing the PPP.^16^ To be correctly implemented, a PPP must be realistic and have inherent value for money for the public partner.^16,17,18^ Public-private partnerships are not equitable to privatisation but are a continuum that links privatisation and public ownership.^19^ It aims to procure services on the populace’s behalf and improve and rehabilitate the public health system.^19^ Both private and public health systems can work more effectively and efficiently by pooling risks, resources and competencies to deliver greater shared public health benefits.^19^ The envisaged benefits, among others, include improved financial risk protection, reduced fragmentation in both funding and provisioning of health services in both public and private sectors, inequalities and improved access to quality healthcare.^20^

Globally, developing nations are popularising PPP for health infrastructure development. The South African government proposes PPP linkages through NHI to enhance service delivery.^19^

In higher-income countries, the private sector plays an essential role in healthcare services delivery, including rehabilitation services,^21,22^ because the public health system is unable to provide the needed health services to all who need it. Aside from infrastructure challenges for rehabilitation in low- and-middle-income countries (LMICs), there is also a problem with human resources capacity for public hospitals or clinics, such as inadequate medical rehabilitation professionals.^6,21,22^ Contrary to the latter, high-level of health professionals are unemployed.^23^ The phasing in of the NHI, with a focus on upgrading public health facilities, producing more health professionals and reducing the relative cost of private health care, is one of 10 critical action points of the national development agenda or policy.^10^

The South African Department of Health (DoH) is supposed to function according to the district health system (DHS) through primary health care (PHC) prioritisation. District health system and PHC facilities are crucial components of a functioning health system in developing countries to promote equal and efficient healthcare.^9^ The inadequacy of the current DHS financing results in the inability of district managers to make effective decisions and the local health benefits of a decentralised health system not being recognised.^24^ The NHI Bill states that the DHS is responsible for forming and collaborating with implementation structures such as District Health Management Offices (DHMOs) and Contracting Units for Primary Health Care Services (CUPS). The DHMOs and CUPS are a bedrock of strategic purchasing that aims to address health system fragmentations, improve healthcare infrastructure and enable accountable and transparent financial management.^11^ A PHC approach through DHS will enhance equitable access to health services and ensure continuity of care in the health system at all stages in the care pathways.^25^

Within this context, PPP may intensify the viability of intermediate care (ICs) or sub-acute care facilities for persons needing extensive district rehabilitation and assist rehabilitation access in rural provinces such as KwaZulu-Natal (KZN). The DoH is incrementally introducing UHC through the NHI; PPP should thus be considered an operational mechanism for the NHI. The UHC successes in countries such as Thailand and Indonesia, nations with low gross national income per capita, have been attributed to sustained federal investment in developing governance to the district structures of healthcare.^11^ Similarly, the Cuban healthcare system, which relies predominantly on CUPS, has shown that the decentralised approach to healthcare allows targeted strategies that address community-specific needs, increase the equity of care and promote managerial flexibility and accountability.^6,26^

This study explored PPP as a form of rehabilitation service provision at the DHS level in anticipation of the NHI in KZN. As a rural province in a developing country, KZN has poor access to and availability of rehabilitation services, particularly in public health.^1,3,4,5^ Chronic shortages of rehabilitation personnel, poor infrastructure, geographically urbanised health institutions, disjointed referral pathways, shifts in global disease patterns and high socio-economic inequality are some reasons for inadequate public health rehabilitation.^1,3,4,5^ An increasing number of governments are electing private sector partnerships to solve the problem of health system defects.^19^

The KZN DoH is supposed to function according to the DHS through PHC. However, rehabilitation services in KZN are mostly institutionally based in acute (district and tertiary) hospitals with services in remote urban areas.^1,2,3,4,5^ The next level of the institution base is community-based rehabilitation (CBR), which therapists and non-therapists^1,3,4,5^ perform. Hospitals cannot discharge a patient directly to their home. Patients may only be partially recovered or their families are not able to care for them while they recover.^25^ After a stroke, for example, the patient might need to adapt to their environment differently in recovery and they need health workers to help them with recovery, such as rehabilitation practitioners, nurses and medical doctors.^6^ Intermediate care facilities differ from hospitals and provide more advanced care than home-based care.^25^ Biopsychosocial rehabilitation services offered by a full complement of rehabilitation disciplines are minimal in rural public health.^1,2,3,4,5^ There is a general lack of the crucial component that plays a critical role in conditions associated with loss of function (CALF) management within the continuum of care, which is ICs in the public healthcare system in KZN. Conditions associated with loss of function conditions are diagnosis-related groupers associated with loss of function, needing designated rehabilitation units or ICs.

As South Africa prepares to implement UHC through the NHI, private sector partnerships are required to solve systemic rehabilitation service provision challenges. Public-private partnerships as a form of infrastructure development are increasingly popular among developing governments.^27,28,29^ The NHI must operate rehabilitation service provision through PPP within the DHS through tertiary district hospitals, IC facilities and community-based care level.

Outcome measure tools are essential to classify patients along the continuum of care and classification of function. In the rehabilitation context, outcome measures are frequently used to assess the characteristics of inpatients and outpatients before any intervention and determine whether patients have made meaningful changes in their recovery process and may influence the intensity and duration of care.^30^ These tools can be jointly used by both private and public sectors on CALF.

Intermediate care facilities or step-down, sub-acute facilities use Multi-disciplinary Teams (MDT) to improve the quality of life for rehabilitation patients discharged from the hospital but require comprehensive therapy to be fully reintegrated into society.^25^ These patients or clients are classified based on low function on discharge from the acute hospitals and needing most or all services of the MDT or CALF. These rehabilitation patients are defined based on diagnosis, surgical procedures and discharge status through diagnostic-related groups (DRGs). The current study explored the rehabilitation perspectives of using PPP to improve access and availability to quality rehabilitation services in KZN in anticipation of South African NHI.

## Research methods and design

### Study design and setting

This study employed a qualitative approach (exploratory qualitative design), using individual interviews and focus group discussions (FGDs) to gather in-depth insights. The research was conducted across three districts within KZN, South Africa: eThekwini Metropolis, King Cetshwayo District Municipality and Amajuba District Municipality. These districts were selected for their unique demographic and infrastructural characteristics, providing a cross-section of urban, peri-urban and rural settings within KZN.

The eThekwini Metropolis, an urban area, is known for its more established healthcare infrastructure but faces significant demand because of its large population. King Cetshwayo district and Amajuba district, with a mix of peri-urban and rural communities, encounter challenges related to limited access to rehabilitation services, with healthcare facilities often located far from remote areas, impacting service delivery and accessibility.

### Sampling

Non-probability maximum variation purposive sampling was used for this study. A total of 57 participants were included, informed by the need to capture diverse perspectives across professions, including physiotherapists, occupational therapists, social workers, audiologists, speech therapists, psychologists, district rehabilitation managers, social development representatives and provincial health representatives (Table 1).

**TABLE 1:** Distribution of the study sample based on Functional Level (*N* = 57).

Functional level	District	Institution	Data collection group	Number of participants
Implementation/service provision	Amajuba	Amajuba District Hospital	Practitioners FGD	15
		Mother and Child Hospital	Practitioners FGD	9
		District Health Office	Rehabilitation Manager Interview	1
Control/monitoring and evaluation	eThekwini Municipality	Victoria Mxenge Hospital	Practitioners FGD	7
Implementation/service provision	King Cetshwayo district	Private Rehabilitation Centre	Practitioners FGD	9
		Ngwelezane Hospital	Practitioners FGD	10
Control/monitoring and evaluation		District Office	Rehabilitation Manager Interviews	2
		Social Development District Office	Social Development Manager Interviews	2
Policy/intelligent development	KwaZulu-Natal province	Department of Health Provincial Office	Provincial Rehabilitation Manager Interviews	2

FGD, focus group discussion.

Participants had to be rehabilitation practitioners and managers from the eThekwini Metropolis, Amajuba district municipality and the King Cetshwayo district municipality. All participant practitioners had to be from the following disciplines: physiotherapy, occupational therapy, nutrition, dietetics, psychology, social work, audiology and speech therapy. KwaZulu-Natal provincial health managers and social development managers were further included.

### Data collection procedure and analysis

Qualitative data were collected through three individual interviews, one dyadic interview, and four FGDs with rehabilitation stakeholders. Participants were recruited over three months (from March 2021 to May 2021) through a purposive sampling process, targeting key rehabilitation professionals and managers in the selected districts. Recruitment involved initial contact via their respective institutions, where information about the study was provided, followed by invitations to participate. Informed consent was obtained before participation, with assurances of confidentiality and the right to withdraw at any time.

Data collection sessions were conducted in locations convenient for participants, creating a comfortable environment to encourage open and honest discussions. Each session, conducted in English as all participants were comfortable expressing themselves in the language, lasted between 30 minutes and 40 minutes. A semi-structured guide ensured consistency across interviews and FGDs while allowing flexibility for participants to share their perspectives on PPPs in rehabilitation services freely. With participants’ permission, all sessions were audio-recorded and transcribed verbatim to ensure accuracy.

Thematic analysis was employed in analysis of the data.^31^ This approach involves familiarising with the data, generating initial codes, searching for themes, reviewing themes, defining and naming themes and finally producing the report. In this study, after initial data familiarisation, we coded transcripts inductively to capture key insights from participants’ responses. These codes were then organised into potential themes relevant to stakeholders’ perspectives on PPPs in rehabilitation services. Themes were iteratively reviewed and refined to ensure they accurately reflected patterned responses within the data, and final themes were defined in relation to the objectives of the study. A theme, in this context, is something that reflects patterned responses within the data.

### Trustworthiness of the study

The study employed several rigorous methods to adhere to the principles of trustworthiness. Transferability was enhanced through detailed, contextual descriptions of the research setting and participant demographics; data collection processes were provided to enable readers to assess the applicability of the findings to other similar contexts, particularly within rehabilitation services and PPP frameworks in similar socio-economic settings. Dependability was addressed by documenting a transparent audit trail of each stage of the research process, including data collection decisions, coding processes and theme development. This approach allowed for an accurate record of methodological decisions and any adjustments made during the study, thereby enabling future researchers to trace and evaluate the consistency of the research approach. Confirmability was ensured through peer debriefing sessions, where preliminary findings and interpretations were reviewed by colleagues familiar with the study’s focus on PPPs in healthcare to reduce researcher bias and ensure objectivity. In addition, reflexive journaling was employed throughout the research process, allowing the lead researcher to document their reflections and potential biases, which helped maintain neutrality in interpreting the data. Credibility was supported by prolonged engagement with participants, achieved through extended interviews and follow-up opportunities, allowed for a deep and authentic understanding of their perspectives. Member checking was also conducted, whereby participants were invited to review and validate the findings, confirming the accuracy and resonance of the interpretations with their views and experiences. These tailored applications of trustworthiness ensured that the study’s findings are robust and reflective of participants’ authentic experiences with PPPs in rehabilitation services.

### Ethical considerations

Ethical approval for the study was secured from the University of KwaZulu-Natal’s Biomedical Research Ethics Committee (BREC), under approval number BREC/00001338/2020. Informed consent was obtained from all participants prior to data collection, in accordance with the Declaration of Helsinki, reinforcing ethical rigour and participant autonomy. Participants were reassured of their right to withdraw from the study without repercussions. Data were securely stored and managed using password-protected electronic databases accessible only to authorised research team members. Hard copies of transcripts were kept in locked cabinets to ensure confidentiality and data security, safeguarding participant information and maintaining the integrity of the research data.

## Results

### Characteristics of the study participants

The participants’ age ranged from 40 to 56 years (Median = 44 years, interquartile [IQR] = 10.5 years) (Table 2). Majority of the participants were female (*n* = 41; 71.9%). A large portion of the sample (*n* = 43; 75.4%) spoke isiZulu as their home language, with the remaining 24.6% spread between English, Afrikaans and other languages. Most (*n* = 49; 85.9%) of the participants held an Honours degree as their highest qualification.

**TABLE 2:** Demographics characteristics of study participants (*N* = 57).

Variable	Statistic	*n*	%
**Age (years)**			
Mean	42.1	-	-
Median	44.0	-	-
Std. deviation	8.6	-	-
Minimum	40.0	-	-
Maximum	56.0	-	-
Interquartile range	10.5	-	-
**Gender**			
Female	-	41	71.9
Male	-	16	28.1
**Home language**			
IsiZulu	-	43	75.4
English	-	9	15.8
Afrikaans	-	3	5.3
Other	-	2	3.5
**Highest qualification**			
Three-year degree	-	3	5.3
Four-year professional degree	-	49	85.9
Postgraduate diploma	-	2	3.5
Master’s degree	-	3	5.3
**Health sector**			
Public	-	39	68.4
Private	-	16	28.1
Hybrid	-	2	3.5

Std., standard.

### Emergent themes

**FIGURE 1:**
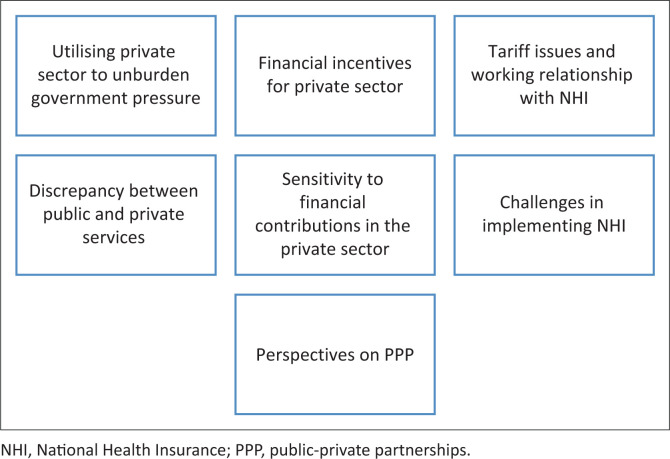
Emergent themes.

The data were reduced to seven themes (Figure 1) and are discussed with supporting verbatim quotes.

### Utilising private sector to unburden government pressure

Stakeholders identified the potential benefits of using PPP to alleviate the burden on government healthcare systems. They suggested that involving private physiotherapists in managing some conditions, such as back issues, could reduce strain on public facilities, improve service availability and enhance the quality of care by leveraging the expertise and resources of the private sector. This approach could make government services more manageable by diverting certain types of cases to private providers:

‘I think you know their plan. I don’t know what’s happening in government now; but they were busy revamping hospitals five years ago. They were revamping hospitals, revamping things you could see their intention was to you know make them part so that everyone will be conditioned to utilise any service because the other thing is to ensure that private physio can see patients with back issues, and then all the back cases can just go there, ankles and you guys only attend maybe those cases who are admitted; operations and things like that. Then it will offload the pressure from government by utilising the private sector.’ (51 year old, female, rehabilitation practitioner, FGD)

### Financial incentives for private sector

Stakeholders were cognisant that participating in PPP could bring financial advantages to the private sector. Stakeholders noted that while a precise embracement model was unclear, the increased patient flow from government referrals could generate more revenue for private practitioners. This financial incentive is viewed as a potential key motivator for private providers to participate in these partnerships:

‘I understand when you are saying it encourages private, I don’t know how they are going to pay, but it makes private get more money and more patients.’ (45 year old, female, rehabilitation practitioner, FGD)

### Tariff issues and working relationship with National Health Insurance

Stakeholders expressed concerns about the potential challenges of tariff standardisation under the NHI system. They worried that differing tariffs between public and private sectors could affect the working relationship with the NHI. Clarity and stability in re-embracement were deemed essential to have their buy-in and, maintain a positive partnership and ensure smooth operation:

‘I just want to add on the NHI thing which I also foresee might be a problem is the tariff issue. Isn’t it that the government must have a standardised tariff? I know that in the private sector there’s COVID and other factors, but I think there might be a challenge. For example, if you normally get a client for 45 hours of treatment and then the government says, ‘I’m giving you 50’, and we’re assigning all the stroke cases to you, that might affect the working relationship with the NHI. I am not a supporter of it, but we will see.’ (41 year old, female, rehabilitation practitioner, FGD)

### Discrepancy between public and private services

A significant discrepancy was noted between public and private rehabilitation services. Rehabilitation practitioners emphasised that the two sectors are fundamentally different, with minimal similarities beyond personnel and human resources. This disparity results in unequal access to quality services, where those unable to afford private care are left with deteriorating public services because of various barriers:

‘There’s such a huge discrepancy between public and private and if someone can come and say here are the similarities, I don’t see them. I believe the only similarities is the physical personnel and the resources in terms of the human resources. You are able to work in private, you can go work in public, other than that there’s absolutely nothing. It’s not equal, not at all; if I can’t go to private then I have to go to public. Which means that automatically I know I will receive deteriorating service because of the huge barriers and hindrances that are.’ (55 year old, female, rehabilitation practitioner, FGD)

### Sensitivity to financial contributions in the private sector

Rehabilitation practitioners acknowledged a heightened sensitivity in the private sector towards patients’ financial contributions. Private healthcare providers are more aware of patients’ financial commitments, such as medical aid contributions, leading to a more personalised and timely approach to care. In contrast, public sector providers often face time constraints, resulting in limited patient interaction:

‘You know when it comes to private and public, with the private patient, just in all honesty you become more sensitive, it’s unfortunate, to the service you provide to them because you are aware of the finances that come into play. You know you’re aware that this person contributes to a medical aid on a monthly basis. You’re also sensitive to the fact that they have limited time with me–I don’t know how to explain it; whereas in government, in public you’re just like ‘ok Mkhulu this is the best 15 minutes I can give you then I have to move on to the next person.’ (47 year old, female, rehabilitation practitioner, FGD)

### Challenges in implementing National Health Insurance

There was scepticism around the feasibility of implementing the NHI in South Africa. Rehabilitation practitioners highlighted challenges such as staffing capacity and the private sector’s ability to meet increased demand for rehabilitation services. These concerns suggest that the NHI may face significant operational and resource allocation hurdles:

‘It’s going to be extremely difficult to push the NHI.’ (41 year old, male, rehabilitation practitioner, FGD)

### Perspectives on public-private partnerships

Provincial health representatives recognised the significance of PPP in enhancing healthcare infrastructure. However, they noted challenges in implementing PPP, especially during the pandemic, as private companies and funders faced resource constraints, limiting their ability to collaborate with the government:

‘Before the pandemic it was very much applicable, but with the pandemic most of these private companies, the funders – because they are also affected – it’s not easy for them to come and partner with government because they also must come with resources.’ (54 year old, female, DoH representative, interview)

Stakeholders also emphasised the importance of including non-governmental organisations (NGOs) in PPP, not just private sector entities. They advocated for the active involvement of NGOs in partnerships to enhance the accessibility and quality of rehabilitation services, suggesting that NGOs should be integral components of collaborative efforts:

‘We should not be limiting the PPP concept to the private sector with the exclusion of NGOs; because NGOs for me ideally should form part of the PPP.’ (56 year old, female, DoH representative, interview)

## Discussion

The findings of the study highlight potential benefits of integrating the private sector to alleviate strain from public healthcare systems. This integrating strategic alignment aims to make government services more sustainable by leveraging private sector expertise and resources, fostering a more balanced healthcare system. A study in South Africa envisaged similar benefits, including improved financial risk protection, reduced fragmentation in both funding and provisioning of the health services in both public and private sectors, reduced iniquities and improved access to quality healthcare.^20^ Similar benefits of PPP have been observed in other studies, such as in India, where private sector involvement improved healthcare delivery and efficiency in public hospitals.^32^

Financial incentives emerged as pivotal in motivating private sector participation in PPP. Stakeholders highlighted that increased patient referrals from government channels could boost revenue for private practitioners, yet concerns lingered over tariff standardisation under the NHI. The partnership has the potential to serve as a vehicle to absorb professionals who struggle with placement post-graduation, yet public health is under-serviced.^23^ Stakeholders stressed the necessity for transparent and stable tariff frameworks to sustain effective public-private collaboration, drawing parallels with challenges observed in other African countries such as Kenya and Nigeria, where tariff disputes and economic instability have hindered the success of PPP in healthcare.^33,34^

The study also identified concerns among practitioners around staffing capacity and resource allocation under NHI. The private sector currently accommodates less than 20% of the population. According to this study’s participants, the lack of consultation with the private sector during the NHI Bill inception has sparked apprehension, particularly concerning infrastructure readiness and the exclusion of medical aid from the funding model. The intention is to create a single framework throughout the Republic for the public funding and public purchasing of healthcare services, medicines, health goods and related products, and to eliminate the fragmentation of healthcare funding in the Republic.^10^ The only mention of the role of the medical aid on the top-up cover, once the NHI has been fully implemented as determined by the Minister through regulations in the gazette, medical schemes may only offer complementary cover to services not reimbursable by the Fund.^10^ These factors contribute to uncertainties about the feasibility of NHI for rehabilitation services, compounded by leadership gaps and scepticism about decision-makers’ grasp of rehabilitation needs.

This is a common viewpoint raised by a previous study stating rehabilitation staff retention and ineffective devolution of management under NHI.^11^ There were also unique or differing viewpoints, including scepticism about decision-makers understanding of rehabilitation, the role of PPP in NHI implementation and the inclusion of NGOs in partnerships. Moreover, stakeholders advocated for broader PPP involvement beyond the private sector, suggesting the inclusion of NGOs and other entities to enhance service delivery. They emphasised the need for well-structured plans to address service availability and patient care challenges within PPP frameworks.

KwaZulu-Natal rehabilitation practitioners highlighted a knowledge gap concerning PPP, underscoring the importance of government-led awareness campaigns and training initiatives. They stressed that policy formation and stakeholder engagement are crucial for effective implementation, pointing to the current limitations of KZN’s PPP definition, which predominantly involves NGOs.^19,35^

As mentioned precedingly, participants expressed a paucity of PPP models within the KZN health system. This knowledge gap requires national and provincial KZN governments to initiate awareness campaigns and training on PPPs for HCWs. Same sentiment was shared by another study which expressed that for any model to be considered for implementation, policy formation, training and inclusion development by related stakeholders is imperative.^11^ This can expand knowledge on the opportunity to leverage PPP to address the faults in the NHI structure. The KZN government’s current PPP definition is restricted to NGOs. KwaZulu-Natal has even set aside funding structure for service over and above their offered services; the invitation is only limited to NGO and/or non-profit organisation (NPO) offering rehabilitation services.^36^ No funding is rewarded if the criteria do not fit within the NPO/NGO bracket.^36^ Government should widen the scope to allow other stakeholders to participate in the delivery of rehabilitation services.^36^ Some policymakers in this study vouched for NGOs. However, the flexibility of PPP, as implied in their definition, allows the inclusion of limited property partners.^36^ The private sector has expertise, financial muscle, technical knowledge and sound infrastructure.^27,29,37,38,39^ As expressed by the participants about the aged and not maintained infrastructure, for example, the government has not addressed such concerns before signing NHI into law. Clearly, it has counted on private sector infrastructure when speaking of quality healthcare, but private healthcare has not been involved in the initial design of policy building up to the NHI Bill. This seems be a challenge on government’s side because unfortunately for the success of the signed Bill, they will need to ensure that they receive a buy in of all stakeholders involved.

Current rehabilitation service access challenges in KZN are immense and systemic for the government to solve alone. For public health to increase access to rehabilitation services in KZN through the NHI, partnerships should be considered as a solution.^27,29,37^ The systemic challenges in the health system are primarily the discrepancies between the private and public sectors. The socio-economic history of South Africa has evolved into a country where quality health care is available to those who can afford it. The cornerstone of the discrepancies is limited financial resources and personnel shortages. The NHI is the government’s attempt to address this current reality.

The DoH’s recent signing into law of the NHI is a noble goal objective of achieving UHC; however, criticism has been levelled against the NHI for being impractical. National Health Insurance is viewed as a social and moral imperative but lacked clarity and insight into the NHI Bill as well as the associated implementation strategies. There has not been an extensive invitation to give stakeholders an opportunity to engage in NHI policy formulation.^11^ Rehabilitation practitioners and managers in KZN expressed concerns about the potential success of the NHI. There were five central negatives participants stressed as reasons for NHI being unachievable. Firstly, a high number of citizens need rehabilitation services versus the systemic shortage of rehabilitation human resources.^12,40^ Secondly, there is a perceived unethical culture of corruption and non-accountability in government.^12,40^ Thirdly, outdated and limited rehabilitation service infrastructure.^3,4,5,41,42^ Fourthly, participants indicated confusion as to how the NHI will function practically. Lastly, participants were pessimistic about the NHI because there is no reliable funding source. After all, private care is sensitive to financial payments. These criticisms allow for the consideration of partnerships to assist the government with successfully implementing the NHI. The health sector requires unison of vision, participation and consensus orientation, transparency and accountability.^11^

Partnerships can accelerate KZN DoH’s actions to decentralise rehabilitation service provision into the DHS.^43^ Intermediate care facilities should be considered key in delivering rehabilitation services and accessible to all communities. Because each district has its unique needs, these facilities should be set based on each district’s health matrix. Each district’s health demographics determine what unique services are required. For example, KZN districts are mostly rural and have fewer industries (stroke and/or medical conditions); industrial areas have industrial health needs, such as injury on duty. Currently, KZN has no meaningful rehabilitation statistic database. There is also a lack of high-level indicators for rehabilitation within national or provincial health monitoring frameworks.^6^ This is because the information gathered from health institutions has no details on the patient’s diagnosis and no system on records for case management and quality assurance.^6^

Public-private partnerships-based DHS rehabilitation will utilise MDT outcome measure tools for effective and seamless referral pathways with MDT.^6,43,44^ Seamline referral pathways can be achieved with a digitised, central, uniform district system.^6,43,44^ Standardised DRGs for rehabilitation clients (stroke, paraplegia, complex fractures, post-surgery, amputees) can create reliable, quality, evidence-based practice.

The KZN government can prepare for rehabilitation service provision through the NHI by partnering with district-health-based, independent, intermediate, MDT, private sector rehabilitation units; this will bring rehabilitation services closer to patients who most need rehabilitation at a local level (PHC).^6,25,40,45^ The private sector demonstrates the early success of referring rehabilitation DRGs to specialised, sub-acute IC facilities.^25,40,45^ Rehabilitation service practice in the private sector proactively determines the path of rehabilitation DRGs from incident to recovery. Intermediate care through PPPs creates resource cross-subsidisation.^6,27,28^ Intermediate care rehabilitation service can be a product purchased by provincial health (DoH) in KZN, with criteria based on disease burden in each district.^25,40,45^

Intermediate care facilities play a crucial role in bridging the gap in the continuum of public health care caused by shortages of practitioners and facilities. These facilities function as step-down, sub-acute units that are more advanced than home-based care options. The NHI underscores ICs’ need to expand access to high-quality rehabilitation services.^6,13,24^ District-based IC units in KZN will deliver rehabilitation services through MDT care, encompassing cognitive, functional and physical expertise. Public-private partnerships can enhance these services to bolster the KZN government’s CBR efforts. Practitioners in IC units can conduct mobile visits to neighbouring district hospitals, providing mentorship and guidance to healthcare workers on clinical expertise, case management and quality assurance. This continuity ensures seamless care transitions for patients across different healthcare settings within the district. Intermediate care units are envisioned as centres of excellence for comprehensive rehabilitation services within local communities. Therapists from these units will assume supervisory and consultant roles, extending their expertise through mentoring and support services.^24,39,44,45^ This integrated approach aims to strengthen rehabilitation care delivery in KZN, promoting better outcomes and equitable access for all residents.

Nevertheless, PPPs are at a nascent stage within the health sector or, indeed developing nations. As such, there is much trial and error. Primarily, PPPs are held with scepticism because of the perceived lack of accountability measures. The nebulous nature of defining a PPP creates confusion between PPPs and ordinary public procurement. Thus, PPPs can be actioned in different methods. Private partners require full transparency on true nature of risks to be shared. The little PPP projects that have occurred primarily on developed countries, indicate that there is sometimes doubt about PPP value for money (VfM); the provision of improved public infrastructure and services at lower cost. Critics question if PPPs are indeed less costly than conventional public spending or if the ‘risks’ of infrastructural development are transferred by government at all.

### Implications for practice and research

The findings of this study emphasise the importance of healthcare policymakers and practitioners prioritising the development and implementation of PPPs and leveraging private sector resources and expertise. Financial incentives and clear tariff structures are essential to fostering effective collaborations between the public and private sectors. Future research should focus on exploring the long-term impacts of PPP on healthcare delivery and developing robust financial models that ensure sustainability and equity in service provision. In addition, there is a need to investigate how NGOs and/or NPOs can be integrated into PPP frameworks to enhance the reach and quality of rehabilitation services. These steps will contribute to advancing healthcare access and delivery in meaningful ways across South Africa.

### Strengths and limitations of the study

One of the primary strengths of this study is the diverse participant cohort, which provided a comprehensive range of perspectives on the use of PPPs in healthcare. The qualitative approach allowed for in-depth exploration of stakeholders’ views, capturing the complexities and nuances of the topic. However, the study also has limitations. The findings are based on participants from KZN, which limits the generalisability of the results to other regions. Additionally, the reliance on self-reported data may introduce bias, as participants might have provided socially desirable responses. Focus group dynamics could have also influenced the findings.

## Conclusion

This study illuminates both the promising benefits and significant challenges associated with leveraging PPP to enhance the accessibility and quality of rehabilitation services in KZN. The findings underscore the imperative of fostering strategic collaborations, ensuring financial transparency and rectifying service disparities to foster an equitable and efficient healthcare framework. Moving forward, future research should prioritise the development of sustainable financial models, explore optimal strategies for integrating NGOs and/or NPOs into PPP initiatives, and resolving operational hurdles inherent in implementing the NHI. By addressing these critical areas, policymakers and practitioners can collectively advance towards realising a more effective and inclusive healthcare system that facilitates improved access to rehabilitation services throughout South Africa.
